# Aptamer-Based Biosensor for Detection of Mycotoxins

**DOI:** 10.3389/fchem.2020.00195

**Published:** 2020-04-21

**Authors:** Xiaodong Guo, Fang Wen, Nan Zheng, Matthew Saive, Marie-Laure Fauconnier, Jiaqi Wang

**Affiliations:** ^1^Key Laboratory of Quality & Safety Control for Milk and Dairy Products of Ministry of Agriculture and Rural Affairs, Institute of Animal Sciences, Chinese Academy of Agricultural Sciences, Beijing, China; ^2^Chimie Générale et Organique, Gembloux Agro-Bio Tech, Université de Liège, Gembloux, Belgium; ^3^Laboratory of Quality and Safety Risk Assessment for Dairy Products of Ministry of Agriculture and Rural Affairs, Institute of Animal Sciences, Chinese Academy of Agricultural Sciences, Beijing, China; ^4^State Key Laboratory of Animal Nutrition, Institute of Animal Science, Chinese Academy of Agricultural Sciences, Beijing, China

**Keywords:** aptamer, biosensor, mycotoxin, detection, food safety

## Abstract

Mycotoxins are a large type of secondary metabolites produced by fungi that pose a great hazard to and cause toxic reactions in humans and animals. A majority of countries and regulators, such as the European Union, have established a series of requirements for their use, and they have also set maximum tolerance levels. The development of high sensitivity and a specific analytical platform for mycotoxins is much in demand to address new challenges for food safety worldwide. Due to the superiority of simple, rapid, and low-cost characteristics, aptamer-based biosensors have successfully been developed for the detection of various mycotoxins with high sensitivity and selectivity compared with traditional instrumental methods and immunological approaches. In this article, we discuss and analyze the development of aptasensors for mycotoxins determination in food and agricultural products over the last 11 years and cover the literatures from the first report in 2008 until the present time. In addition, challenges and future trends for the selection of aptamers toward various mycotoxins and aptasensors for multi-mycotoxins analyses are summarized. Given the promising development and potential application of aptasensors, future research studies made will witness the great practicality of using aptamer-based biosensors within the field of food safety.

## Introduction

**Figure 1:**
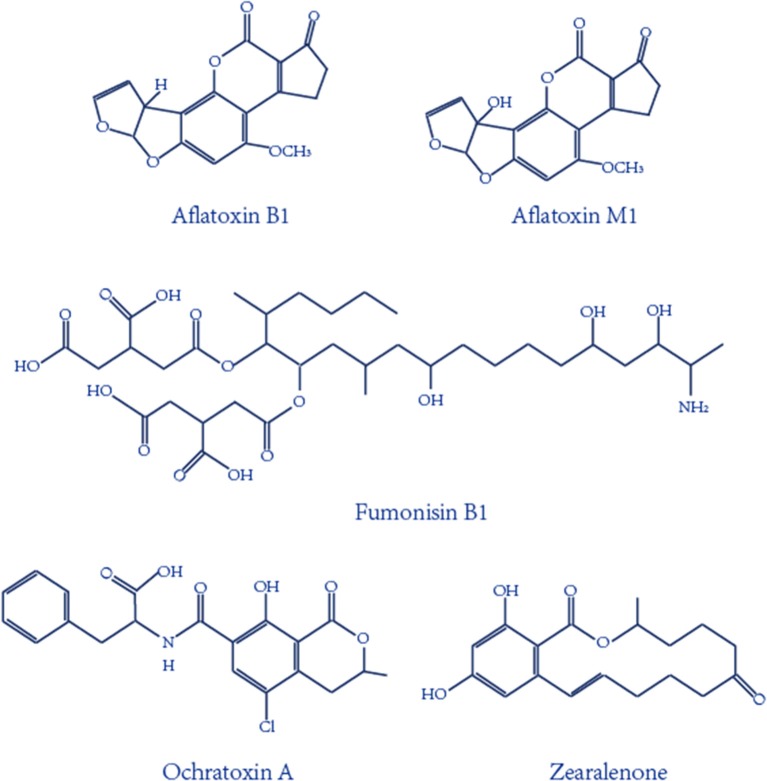
Chemical structures of the important mycotoxins.

Mycotoxins, one of the most important and toxic contaminants in food and agricultural products, are secondary metabolites produced mainly by various molds (Atar et al., 2015; Mata et al., 2015; Zhu et al., 2015). Hundreds of mycotoxins have been identified, and the main ones are aflatoxins (AF), ochratoxins (OT), fumonisins (F), and zearalenone (ZEN), as seen in Figure 1. Aflatoxin B1 and aflatoxin M1, the most toxic mycotoxins, have been designated as a group 1 carcinogen by the International Agency for Research on Cancer (IARC) of the World Health Organization (WHO), and ochratoxin A has been classified as a group 2B carcinogen by IARC (International Agency for Research on Cancer (IARC), 1993, 2002; O'Brien and Dietrich, 2005). Given the serious toxicity effect of mycotoxins on animals and humans, the European Commission set the maximum contamination AFB1 level to 2 μg kg^−1^ for all cereals and cereal-derived products for food safety (Commission, 2010). In addition, the European Union has regulated a maximum tolerance level for AFM1, set to 0.050 μg kg^−1^ for an adult and a lower level, 0.025 μg kg^−1^, for child and infant consumption (Commission Recommendation, 2006). Taking the high toxicity and low permissible limits into consideration, rapid, low-cost, sensitive analytical strategies for the detection of mycotoxins are vitally important and a necessity.

**Figure 2:**
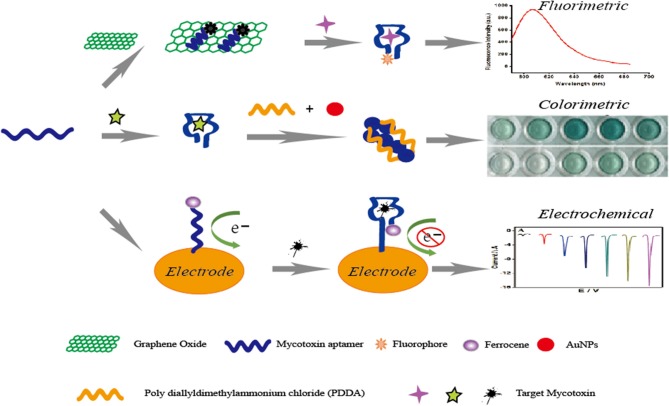
Principle illustration of fluorescent, colorimetric and electrochemical aptasensors for the detection of small molecule mycotoxins.

Confirmatory and quantitative approaches for the detection of mycotoxins mainly include thin layer chromatography (TLC) (Var et al., 2007), high-performance liquid chromatography (HPLC) (Herzallah, 2009; Wang et al., 2012; Yazdanpanah et al., 2013; Lee and Lee, 2015; Mao et al., 2015; Pietri et al., 2016), and liquid chromatography coupled with mass spectrometry (LC–MS) (Corcuera et al., 2011; Abia et al., 2013; Warth et al., 2013). However, expensive and special instruments, complicated pretreatment, and professional personnel are required for the use of these typical equipment methods (Shim et al., 2007). In the meantime, antibody-based immunoassays have been developed for mycotoxin detection, and these include enzyme-linked immune sorbent assays (ELISAs) and immunosensors methods (Piermarini et al., 2007; Li et al., 2009; Parker and Tothill, 2009; Kav et al., 2011; Bacher et al., 2012; Mozaffari Nejad et al., 2014; Park et al., 2014; Sheng et al., 2014; Vdovenko et al., 2014; Xu et al., 2014; Anfossi et al., 2015). Though the immunoassays have the advantage of high specificity, the high cost and storage stability of antibodies limits the application of these rapid analysis procedures. Aptamers, an alternative molecule recognition element to antibodies, are single-stranded (ss) DNA or RNA oligonucleotides that can form aptamer/target complexes with very strong affinity and specificity via the conformational change. The advantages of aptamers are compared to antibodies in Table 1. With these advantages, aptamer-based biosensors have been widely introduced for the detection of mycotoxins like AFB1 (Evtugyn et al., 2013; Shim et al., 2014; Castillo et al., 2015; Seok et al., 2015; Wang et al., 2016a), AFM1 (Nguyen et al., 2013; Istamboulie et al., 2016), OTA (Kuang et al., 2010; Guo et al., 2011; Yang et al., 2013), FB1 (Wu S. et al., 2012; Wu et al., 2013), especially based on fluorescent, colorimetric, and electrochemical aptasensors (Figure 2). However, ultrasensitive approaches are difficult to develop via simple aptasensor recognition. Therefore, a series of novel aptasensors with signal amplification and enhancement have been introduced for mycotoxins (Patolsky et al., 2002; Pavlov et al., 2004; Weizmann et al., 2006; Yang et al., 2007; Deng et al., 2009; Guo X. et al., 2014; Wu et al., 2017), which can meet the requirement of the low maximum contamination level set by many countries and organizations.

**Table 1:** Comparison of the properties between antibody and aptamer.

**No**.	**Characteristics**	**Antibody**	**Aptamer**
1	Molecular size	Big molecular	Small molecular
2	Screened conditions	Screened under physiological conditions	Screened and chemical synthesis *in vitro*
3	Sensitivity to temperature	Sensitive to temperature, short storage time	Stored and transported at room temperature
4	Stability	Temperature-induced denaturation is irreversible	Temperature-induced denaturation is reversible
5	Cost	Long preparation time with high cost	Short preparation time with low cost
6	Immunogenicity	Strongly immunogenic	No obvious immunogenicity
7	Targets	Limited target substances	Wide range of target substances
8	Modifiability	Lose affinity to target with labels	Keep original biological activity with labels
9	Cross-reactivity	Unable to separate cross-reactive substances	Can separate structural analogs or cross-reactive substances

## Aptamers Selection

Aptamers are selected by systematic evolution of the ligand by the exponential enrichment process (SELEX) *in vitro*, which was first introduced against T4 DNA polymerase (Tuerk and Gold, 1990) and organic dyes (Ellington and Szostak, 1990). In recent years, a series of SELEX-based techniques have been developed for the selection of aptamers against various targets, mainly including cell-SELEX, capillary electrophoresis-SELEX (CE-SELEX), immunoprecipitation-coupled SELEX (IP-SELEX), atomic force microscopy SELEX (AFM-SELEX), and artificially expanded genetic information system-SELEX (AEGIS-SELEX). As shown in Table 2, characteristics, advantages, and disadvantages are summarized and discussed.

**Table 2:** Comparison of the current SELEX-based aptamer selection techniques (Zhang et al., 2019).

**SELEX**	**Key points**	**Advantages**	**Disadvantages**
Cell-SELEX	Aptamers selection for whole live cells	Aptamers for molecules in their native state. Cell surface has many potential targets. No protein purification	Costly. For cell surface targets. Time consuming. Post SELEX identification of the target required
CE-SELEX	Electrophoretic mobility-based ions separation	Rapid. Only 1–4 rounds of selection. Reduced non-specific binding. No target immobilization	Not suitable for small molecules. Expensive equipment required
IP-SELEX	Includes immunoprecipitation	For target proteins under physiological conditions. Increased affinity and specificity	Time-consuming
AFM-SELEX	Employs AFM for 3D image of sample surface	Rapid. Only 3–4 rounds required. Increased aptamer affinity	Expensive equipment required. Target and aptamers immobilization required
M-SELEX	Microfluidic system-based SELEX	Rapid. Automatable. For small molecules. Highly efficient (required only small amounts of reagents)	Low purity of aptamers. Target immobilization required
AEGIS-SELEX	Libraries with the artificially expanded genetic code	Increased aptamer specificity	Poor recognition of the unnatural bases by natural DNA polymerases
Capture-SELEX	Library is immobilized on a support	For small molecules. For structure-switching aptamers. No target immobilization	Partial oligonucleotides from the library might be not selected

## Aptasensor for the Analysis of Ochratoxin A

Ochratoxins are one important type of mycotoxin, and they are mainly produced by several fungi, such as *Aspergillus ochraceus* and *Penicillium verrucosum* (Liu et al., 2015). Of the several subtypes of ochratoxins, ochratoxin A (OTA) is the most common and has been designated as a possible human carcinogen by IARC (Lv et al., 2016). Researchers have paid much attention to studies on OTA in recent years, owing to its widespread occurrence and extraordinary toxic reactions in animals and humans. The first aptamer, the minimal one of the selected sequences, has the highest affinity to OTA. The dissociation constant is 200 nM. Since this aptamer specific to OTA was reported by Cruz-Aguado in 2008 (Cruz-Aguado and Penner, 2008), large numbers of novel aptasensors were developed for OTA analysis in various food products, including fluorescent, colorimetric, and electrochemical aptasensors, as well as some methods based on nanomaterials. The recent literature on aptasensors for the analysis of ochratoxin A for food safety are illustrated in Table 3. In addition, these articles have been analyzed in more detail for each target group.

**Table 3:** Summary of aptasensors for the analysis of ochratoxin A.

**Mycotoxins**	**Method**	**Principle**	**Detection range**	**LOD**	**Sample**	**Reference**
OTA	Fluorescent aptasensor	Single-walled carbon nanotubes (SWNTs) as quencher	25–200 nM	24.1 nM	Beer	Guo et al., 2011
OTA	Fluorescent aptasensor	PVP-protected graphene oxide as quencher	50–500 nM	21.8 nM	Red wine	Sheng et al., 2011
OTA	Fluorescent aptasensor	Target-induced conformational change signaling aptamer	1–100 ng mL^−1^	0.8 ng mL^−1^	Corn	Chen et al., 2012
OTA	Fluorescent aptasensor	Fluorescent DNA and silver-nanocluster (AgNCs)	0.01–0.3 ng mL^−1^	2 pg mL^−1^	Wheat	Chen et al., 2014
OTA	Fluorescent aptasensor	Carboxy-modified fluorescent Particles	0.1–150 nM	0.005 nM	Beer	Hayat et al., 2015
OTA	Fluorescent aptasensor	Aptamer-conjugated magnetic beads (MBs) and CdTe quantum dots (QDs)	0.015–100 ng mL^−1^	5.4 pg mL^−1^	Peanut	Wang et al., 2015
OTA	Fluorescent aptasensor	Hybridization chain reaction (HCR)	1.0–20 pM	0.1 pM	Corn	Wang et al., 2016b
OTA	Colorimetric aptasensor	Unmodified gold nanoparticles (AuNPs) indicator	20–625 nM	20 nM	–	Yang et al., 2011
OTA	Colorimetric aptasensor	Target-reactive aptamer-cross-linked hydrogel	0–100 nM	1.27 nM	Beer	Liu et al., 2015
OTA	Colorimetric aptasensor	Cationic polymer and AuNPs	0.05–50 ng mL^−1^	0.009 ng mL^−1^	liquor	Luan et al., 2015
OTA	Colorimetric aptasensor	Aptamer-controlled growth of Au NPs	–	1 nM	Red wine	Soh et al., 2015
OTA	Colorimetric aptasensor	Au@Fe3O4 NPs	0.5–100 ng mL^−1^	30 pg mL^−1^	Peanut	Wang C. et al., 2016
OTA	Electrochemiluminescent biosensor	N-(4-aminobutyl)-N-ethylisoluminol and AuNP-modified gold electrode	0.02–3.0 ng mL^−1^	0.007 ng mL^−1^	Wheat	Wang et al., 2010
OTA	Electrochemical aptasensor	Aptamer modified gold electrode	0.1–1,000 pg mL^−1^	0.095 pg mL^−1^	Red wine	Wu J. et al., 2012
OTA	Electrochemical aptasensor	Gold electrode coupled with silver nanoparticles	0.3–30 nM	50 Pm	Beer	Evtugyn et al., 2013
OTA	Electrochemical aptasensor	Rolling circle amplification (RCA)	0.1–5,000 pg mL^−1^	0.065 pg mL^−1^	Wine	Huang et al., 2013
OTA	Electrochemical aptasensor	Au NPs and methylene blue	2.5–2,500 pM	0.75 pM	Red wine	Yang et al., 2014
OTA	Electrochemical aptasensor	Exonuclease-induced recycling amplification	0.01–1.0 ng mL^−1^	0.004 ng mL^−1^	Corn and Oat	Tan et al., 2015
OTA	Electrochemical aptasensor	Nanocomposites of AuNPs	0.2–4,000 pg mL^−1^	0.07 pg mL^−1^	–	Hao et al., 2016
OTA	Chemiluminescence aptasensor	HRP-mimicking DNAzyme (HRPzyme)	0.1–100 ng mL^−1^	0.22 ng mL^−1^	Coffee beans	Jo et al., 2016
OTA	Electrochemical aptasensor	Exonuclease (Exo) III-assisted recycling amplification	0.001–0.5 ng mL^−1^	0.58 pg mL^−1^	Wheat	Liu et al., 2016

### Fluorescent Aptasensor for OTA

**Figure 3:**
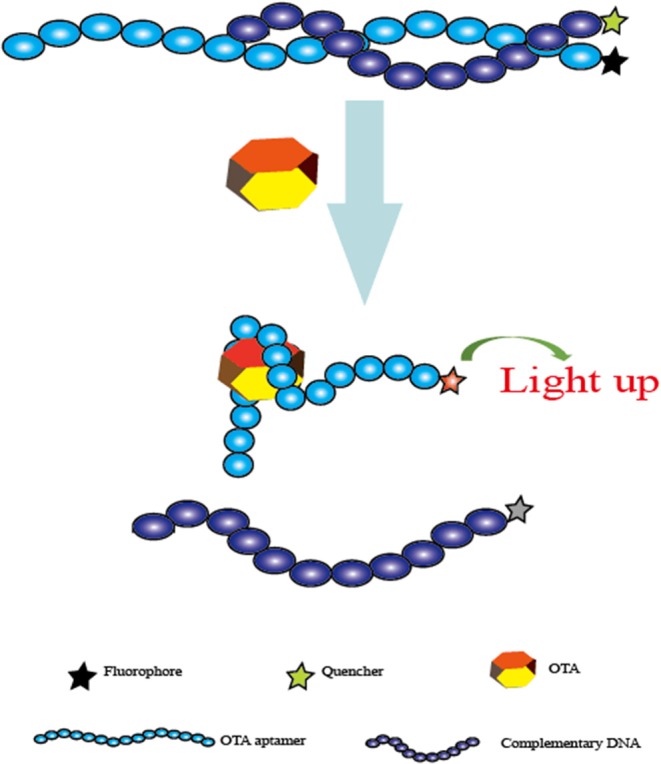
Schematic representation of the fluorescent aptasensor for OTA determination based on the conformational change of aptamer.

First, Guo et al. developed a sensitive and selective aptasensor for fluorescent detection of OTA. Single-walled carbon nanotubes (SWNTs) were employed to quench the fluorescence signal produced by carboxyfluorescein-labeled aptamers. With the presence of OTA, the spatial structure change of the specific aptamer leads to the separation of the SWNTs and the aptamer, and the fluorescence is therefore detected. The fluorescence signal has a good linear relationship with concentrations in a range from 25 to 200 nM and a detection limit of 24.1 nM (Guo et al., 2011). In addition, this aptasensor is successfully used for OTA determination in real beer samples. In the same year, another fluorescent aptasensor was introduced by the same research team via the use of graphene oxide as a fluorescence quencher (Sheng et al., 2011). In this sensing platform, a linear response of 50–500 nM was obtained between the fluorescence intensity and OTA levels with a detection limit of 21.8 nM. More importantly, the limit detection could be lowered by two orders of magnitude by using PVP-coated graphene oxide. Similarly, this current aptasensor was also validated for OTA detection in red wine samples. Based on the similar fluorescence response and fluorescence quench schemes, Chen et al. reported a rapid and feasible aptasensor for OTA determination in corn samples (Chen et al., 2012). In this sensing platform (Figure 3), the fluorophore was used to label the aptamer, while the quencher moiety was employed to label the complementary DNA to quench the fluorescence. When there was no OTA, the hybridization reaction between the complementary DNA and the aptamer led to the close distance of these two moieties, and the fluorescence signal was effectively quenched. Upon OTA addition, the production of the aptamer/OTA complex resulted in the separation of the complementary DNA, and the fluorescence was thus recovered. A good linear response between the fluorescent change and OTA levels was obtained and ranged from 1 to 100 ng mL^−1^ with a detection limit of 0.8 ng mL^−1^. More importantly, the whole analysis process of this method was completed within 1 min. Therefore, this was another fluorescent aptasensor for rapid determination of OTA with high efficiency.

Nanomaterials were also used to construct biosensors with several modifications to produce fluorescence. One of those aptasensors was introduced for the detection of OTA. In this research, magnetic beads, the fluorescence characterization of silver nanocluster DNA, and the spatial conformational change of the aptamer were adopted. An ultrasensitive method was achieved with the limit detection (2 pg mL^−1^). Additionally, the practical analysis was successfully completed on wheat samples for OTA detection. This aptasensor might provide a promising sensing platform for OTA due to its unique advantages of being low in cost, rapid, portable, selective, and ultrasensitive (Chen et al., 2014). Based on the same magnetic bead separation method, Akhtar et al. reported a generic fluorescent aptasensor to detect OTA. In this sensing strategy, a carboxy-labeled aptamer was adopted to signal produce element, the magnetic beads were employed to separate unbound portions. The high sensitivity of this aptasensor was determined with a limit detection (0.005 nM). Moreover, this proposed method was successfully applied to beer samples for the analysis of OTA (Hayat et al., 2015).

Additionally, another one-step fluorescent aptasensor was illustrated for OTA determination. The specific aptamer for OTA was employed as a target recognition probe, CdTe quantum dots (QDs) were used as a label, and magnetic beads acted as the separation support. Upon OTA addition, the production of the aptamer/OTA complex and magnetic separation resulted in a significant fluorescence intensity enhancement. More importantly, a wide-range response between the fluorescence signal and OTA concentrations (from 15 pg mL^−1^ to 100 ng mL^−1^) was achieved, and the limit of detection was 5.4 pg mL^−1^. Therefore, this developed sensing platform might represent a potential strategy for OTA routine controls for food safety (Wang et al., 2015). Finally, the hybridization Chain Reaction (HCR), an important signal-enhancement strategy, has seen widespread application for the analysis of DNA, proteins, metal ions, viruses, cancer cells, as well as mycotoxins. An ultrasensitive aptasensor was developed for OTA determination via the HCR technique. In this sensing platform, the perylene derivative was conjugated and employed as the fluorescence element. DNA concatamers were obtained through the HCR strategy, which caused the aggregation of the perylenediimide probe and subsequent signal enhancement. Under the optimized conditions, the fluorescence signal was established to have a good linear relationship with the targeted OTA concentrations. It ranged from 1.0 to 20 pM with a detection limit of 0.1 pM, indicating that this current aptasensor was highly sensitive during the analysis of the OTA. Moreover, the practicality of this sensing strategy was validated through the successful detection of OTA levels in corn samples (Wang et al., 2016b).

### Colorimetric Aptasensor for OTA

**Figure 4:**
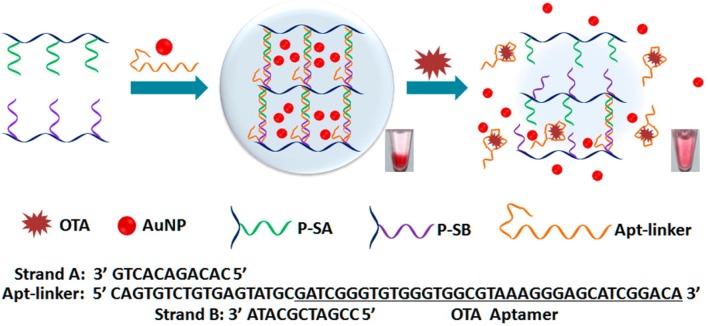
Principle illustration of colorimetric aptasensor for detection of OTA via AuNPs encapsulated DNA hydrogel. Reprinted from Liu et al. (2015) with permission.

In addition to fluorescent aptasensors, colorimetric aptasensors are also widespread techniques for the analysis of mycotoxins because of they are simple and rapid to use and are without the requirement of complicated instruments. A color change was produced as a result of the aggregation of gold nanoparticles (AuNPs) in the salt presence. Yang et al. developed a colorimetric aptasensor for OTA determination, which employed the advantages of the specific aptamer and AuNPs (Yang et al., 2011). Upon the OTA addition, structural change to the G-quadruplex of the aptamer from random coil to G-quadruplex resulted in the separation of the aptamer from AuNPs, leading to AuNPs aggregation under the addition of salt and a subsequent color change. The absorbance values presented a good linear relationship with OTA concentrations ranging from 20 to 625 nM. Its detection limit was 20 nM. Using the same AuNPs colorimetric indicators, a novel visual aptasensor was introduced for the visual analysis of OTA via the synthesis of DNA hydrogels. This technology has been widely applied for the analysis of proteins (Zhang et al., 2013), ions (Dave et al., 2010; Lin et al., 2011; Guo W. et al., 2014), nucleic acids (Sun et al., 2014), as well as small molecules (Zhu et al., 2010; Yan et al., 2013). In this design, as depicted in Figure 4, the DNA hydrogels are obtained through linkage between the aptamer of OTA and two polymer single-stranded DNAs. With the addition of OTA, the aptamer/OTA complex formation induced dissociation of the DNA hydrogels. Subsequently, the AuNPs were released for the colorimetric detection of OTA levels. The detection limit was determined to be 1.27 nM by signal amplification strategy. Therefore, this proposed sensing strategy represented a novel and portable method for the analysis of OTA to ensure food safety (Liu et al., 2015). Moreover, Luan et al. reported a sensitive and selective aptasensor for OTA determination via aggregation of AuNPs as the colorimetric generator induced by poly diallyldimethylammonium chloride (PDDA). Upon the optimal conditions, its detection limit was obtained down to 0.009 ng mL^−1^, which demonstrated that this sensing platform provided a promisingly simple and sensitive platform for the rapid analysis of OTA (Luan et al., 2015).

Under the normal circumstances, the coexistence of multiple mycotoxins is a very common phenomenon in food and agricultural products. High-throughput screening and analysis of multiple mycotoxins will play an important role in food safety. Based on the recognition reaction of the aptamer and the colorimetric response of AuNPs, a rapid and sensitive aptasensor for the analysis of multiple small molecules including OTA was introduced. Upon the addition of target, the interactions between the aptamer and targets caused the dissociation of the aptamer from the surface of AuNPs, leading to the color change of AuNPs. The limits of detection were 1 nM for OTA, 0.2 nM for 17β-estradiol, and 1 nM for cocaine. Therefore, the novel aptasensor became a potential strategy for high-throughput application for multiple mycotoxins detection by the simple replacement of an aptamer for different mycotoxins (Soh et al., 2015). In addition, Au@Fe_3_O_4_ NPs were obtained through AuNPs being functionalized by Fe_3_O_4_ NPs, and the Au@ Fe_3_O_4_ NPs activity was thus increased. The aptamer of OTA was labeled to the magnetic beads, while its complementary single-stranded DNA was modified on Au@ Fe_3_O_4_ NPs. Upon the addition of OTA, the recognition between the aptamer and OTA caused the release of ssDNA-labeled Au@ Fe_3_O_4_ NPs, which could induce a color change to a blue solution. Its detection limit was down to 30 pg mL^−1^, demonstrating that this sensing strategy was a novel and sensitive approach for OTA determination for food safety via the application of peroxidase-like activity of AuNPs (Wang C. et al., 2016).

### Electrochemical Aptasensor for OTA

The immobilization of aptamers on the surface of transducer substrates is an important function in the construction of electrochemical aptasensors. The transducer substrates mainly consisted of gold and carbon-based electrodes. A series of electrochemical aptasensors were developed for OTA analysis in food and agricultural products, including Electrochemical Impedance Spectroscopy (EIS), Differential Pulse Voltammetry (DPV), Cyclic Voltammetry (CV), Linear Sweep Voltammetry (LSV), Square Wave Voltammetry (SWV), Field Effect Transistor, and potentiometry. Electrochemical aptasensors have attracted great interest from researchers owing to their advantages of being rapid, portable, and of low cost, and they also have high sensitivity, selectivity, as well as efficiency. Firstly, based on the AuNPs-modified gold electrode and luminescence-labeled specific aptamer, Wang et al. successfully carried out an electrochemiluminescent aptasensor method for OTA determination in wheat samples. The luminescence-labeled aptamer was employed as the indicator of the sensor, and the complementary DNA modified to gold electrode was hybridized with the aptamer. Upon the addition of OTA, the structural change of the aptamer caused the separation of the luminescence-labeled aptamer from the complementary DNA-modified gold electrode, resulting in the decrease of electrochemiluminescence (ECL) signals. In optimized conditions, a good linear response was observed between the ECL signals and OTA concentrations ranging from 0.02 to 3.0 ng mL^−1^, and its detection limit was 0.007 ng mL^−1^. Therefore, this current method offered a promising sensing strategy for the analysis of OTA for food safety (Wang et al., 2010). Then, by using the same gold electrode, an electrochemical aptasensor with high sensitivity was introduced for OTA detection (Wu J. et al., 2012). The aptamer labeled with thiol and methylene blue was used as the molecule recognition probe. Under the addition of target OTA, an aptamer/OTA complex was formed owing to the interaction of the aptamer and OTA, which suppressed the transfer of the electron. In the optimized conditions, the change in the electrochemical signal saw a good linear response when compared to the OTA levels ranging from 0.1 to 1,000 pg mL^−1^. Its detection limit was determined to be 0.095 pg mL^−1^. Moreover, the proposed electrochemical aptasensor was successfully used for the OTA analysis in red wine samples with satisfying recoveries. This rapid approach represented a potential tool for on-site analysis of OTA.

Similarly, based on the gold electrode and recognition of the aptamer, another electrochemical aptasensor was presented for OTA determination (Evtugyn et al., 2013). In this study, the gold electrode was modified using silver nanoparticles and electropolymerized neutral red, and the aptamer was thiolated to the surface of the silver nanoparticles. With the addition of OTA, an aptamer/OTA complex was produced, which caused the change of charge transfer resistance subsequently determined via EIS in the addition of ferricyanide ions. The limit of detection was calculated to be 0.05 nM. In addition, the method validation was confirmed through the analysis of this electrochemical aptasensor for OTA analysis in beer samples with satisfactory recoveries.

**Figure 5:**
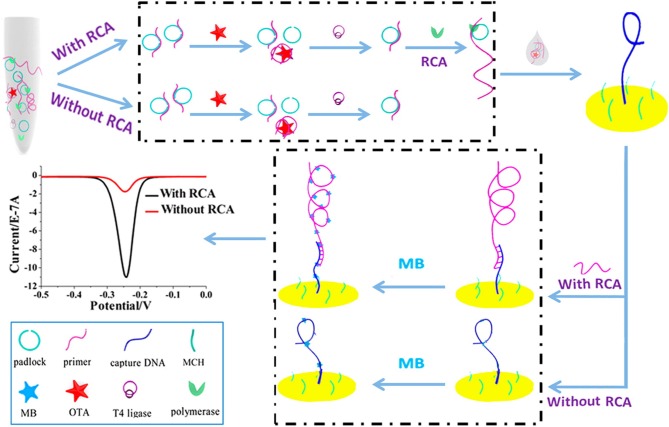
Principle diagram of electrochemical aptasensor for OTA analysis based on RCA signal amplification. Reprinted from Huang et al. (2013) with permission.

It is important to note that many government regulators and countries have set a very low level of maximum residue of OTA in food and agricultural products due to its serious toxicity to humans and animals. The signal enhancement techniques are a requirement when using this technology. The rolling circle amplification (RCA) technique was used to improve the method sensitivity of electrochemical sensors. Huang et al. developed an electrochemical aptasensor for OTA determination on the basis of the specific recognition of the aptamer and the signal amplification of RCA (Figure 5). The RCA primer consisted of two DNA sequences, including the aptamer, and the complementary DNA modified on surface of the electrode. With the OTA presence, RCA was suppressed because of the recognition between OTA and its aptamer, leading to a decreasing signal from the electrochemical aptasensor. In optimized conditions, its detection limit was obtained at 0.065 pg mL^−1^. In addition, the feasibility and practicability were validated for OTA determination in wine samples (Huang et al., 2013).

To improve detection sensitivity, a novel duplicate and signal-enhanced electrochemical aptasensor was presented for OTA detection in red wine samples (Yang et al., 2014). In this research, four DNA sequences were designed and synthesized. DNA3-GNPs/DNA2/DNA1-Au were modified to the surface of the electrode via strand hybridization for the first signal enhancement with the addition of methylene blue. Meanwhile, the DNA4, partially complementary to DNA3, was able to offer several anchoring sites for MB due to its rich G bases. The duplicate signal amplification was thus obtained. A good electrochemical signal response was achieved against the concentrations of OTA in the 2.5 pM to 2.5 nM range, and the detection limit was 0.75 pM.

However, the novel electrochemical aptasensors were also achieved via immobilization-free aptamers to the surface of electrode. The complementary DNA, labeled with MB, was adopted as the probe DNA of the electrochemical aptasensor. Since DNA contains negative charges, the hybridization duplex of the aptamer and its complementary DNA was not diffused to the surface of the negatively charged electrode due to the repulsion reaction. Upon the presence of OTA, the conformational changes of the aptamer led to the release of a DNA probe, subsequently digested by the exonuclease. As the DNA probe contains a low amount of negative charge, it spreads to the surface of the electrode, and the electrochemical signal is thus increased. On the other hand, the target OTA was released for the next cycle to obtain the detection sensitivity. Based on the electrochemical method, an ultrasensitive aptasensor was introduced with a detection limit of 0.004 ng mL^−1^ (Tan et al., 2015), demonstrating that this aptasensor approach provided a potential analysis strategy for various contaminants. Meanwhile, Hao et al. reported an electrochemical aptasensor for OTA analysis via AuNPs-functionalized iron oxide magnetic nanomaterials and graphene/AuNPs nanoparticles. A good linear response was obtained between the electrochemical aptasensor and the target OTA levels in the range of 0.2 pg mL^−1^-4 ng mL^−1^. Its detection limit was established at 0.07 pg mL^−1^ (Hao et al., 2016). The proposed aptasensor offered a promising strategy for the analysis of mycotoxins for food safety.

**Figure 6:**
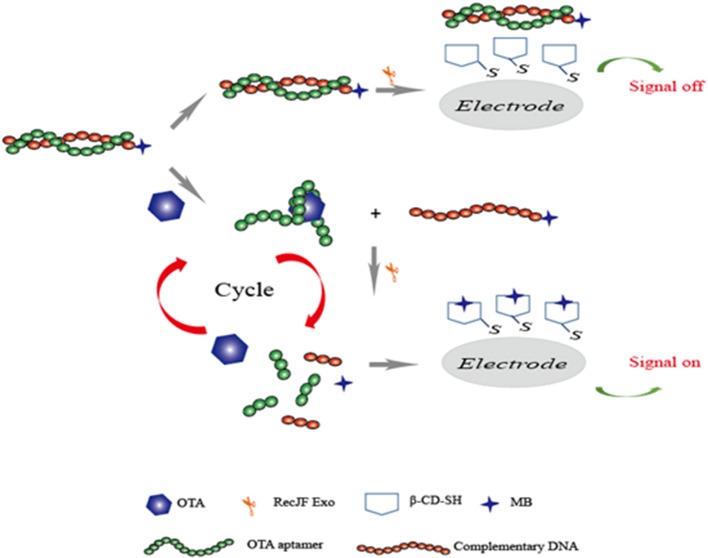
Sensing strategy of electrochemical aptasensor for detection of OTA based on exonuclease-assistant signal amplification.

In addition, chemiluminescence techniques were also widely employed for food safety research owing to its simple, low-cost, and sensitive characteristics. Chemiluminescence resonance energy transfer (CRET) is a process of energy conversion between a chemiluminescent donor and an acceptor. Since no external light source was needed, low background signals were produced in the chemiluminescence methods. By taking advantages of chemiluminescence, Jo et al. introduced a chemiluminescent aptasensor for OTA determination in coffee. In this study, the specific aptamer to OTA was synthesized with the 5′-modification of DNAzyme and 3′-modification of dabcyl, which was used as the quencher of the CRET aptasensor. Upon the OTA and hemin addition, the complex produced by G-quadruplex/OTA led to the aptamer approaching the hemin, and CRET caused the quenching of the chemiluminescence signals between luminol and dabcyl. The chemiluminescence signals had a better response than the OTA concentrations ranging from 0.1 to 100 ng mL^−1^. Its detection limit was established to be 0.22 ng mL^−1^ (Jo et al., 2016). Recently, based on a similar principle via the chemiluminescence method and the formation of G-quadruplex, a novel chemiluminescence strategy-based aptasensor for OTA determination was developed with higher sensitivity (LOD = 0.07 ng mL^−1^). The triple-helix aptamer probe (TAP) consists of the aptamer, two DNA stems, and a signal probe. In the OTA addition, the production of the aptamer/OTA complex led to the release of the signal probe from TAP. With the presence of hemin and potassium ions, a conformational change of signal probe to G-quadruplex could catalyze luminol, and the chemiluminescence signal was then obtained (Wang et al., 2019a). A series of studies have focused on the enhancement of methods-detection sensitivity via the application of exonuclease III (Exo III), which can effectively digest double-stranded DNA. The recycling amplification was therefore achieved without any specific recognition elements. A rapid and simple electrochemical aptasensor with high sensitivity was introduced for OTA determination in wheat samples. In this study, the specific aptamer was adopted as a molecule recognition sequence, and Exo III was employed to achieve the recycling amplification. With the addition of target OTA, the recognition between aptamer and OTA resulted in the release of complementary DNA and subsequent digestion by Exo III. A good linear response was detected between the electrochemical signals and OTA concentrations in the range of 0.001 to 0.5 ng mL^−1^ with a detection limit of 0.58 pg mL^−1^ (Liu et al., 2016). Based on the same exonuclease signal amplification technique, another research group developed an electrochemical aptasensor for sensitive detection of OTA in red wine. In this study, as shown in Figure 6, complementary DNA (cDNA) of the OTA aptamer was modified with methylene blue (MB). The aptamer was hybridized with cDNA to form a dsDNA complex. In the presence of OTA, the aptamer/OTA complex was formed with a G-quadruplex structure, and the cDNA was then released from the dsDNA to the surface of the electrode. Subsequently, the aptamer was digested partially by RecJf exonuclease. Thus, the target OTA was released for the next cycle. Ultimately, an exonuclease signal amplification aptasensor was achieved with satisfied detection range (from 10 pg mL^−1^ to 10 ng mL^−1^) and LOD (3 pg mL^−1^) (Wang et al., 2019b).

## Aptasensor for the Analysis of Aflatoxins

Aflatoxins (AFs), one of the most toxic mycotoxins, are secondary metabolites produced by a variety of molds that mainly include *Aspergillus flavus* and *Aspergillus parasiticus*. AFs occur predominantly in feeds and agricultural products, like peanuts, cereals, corn, as well as the tree nuts. Among the several kinds of AF (including B1, B2, M1, M2, G1, and G2), AFB1 and AFM1 are the most toxic ones and have been classified as group 1 carcinogenic compounds by IARC (International Agency for Research on Cancer (IARC), 2002). Therefore, many countries and organizations have established a maximum contamination level of these toxic mycotoxins for food safety (Commission, 2010). In 2012, the high affinity aptmer to AFB1 was first selected by Neoventures Biotechnology Inc. (Canada) (Patent:PCT/CA2010/001292, Patent:PCT/CA2010/001292). This aptamer specific to AFM1 was selected and characterized by Malhotra et al. (2014). With the advantages of these aptamers for aflatoxins, aptamer-based biosensors were successfully developed for aflatoxins determination and have been studied in depth in the literature (Table 4).

**Table 4:** Summary of aptasensor for aflatoxins analysis and fumonisins and zearalenone.

**Mycotoxin**	**Method**	**Principle**	**Detection range**	**LOD**	**Sample**	**Reference**
AFB1	Fluorescent aptasensor	CdTe quantum dots and graphene oxide	3.2 nM−320 μM	1.0 nM	Peanut oil	Lu et al., 2014
AFB1	Fluorescent aptasensor	Nanographene oxide and nuclease	1.0–100 ng mL^−1^	0.35 ng mL^−1^	Corn	Zhang et al., 2016a
AFB1	Colorimetric aptasensor	Peroxidase mimicking DNAzyme activity	0.1–10,000 ng mL^−1^	0.1 ng mL^−1^	Corn	Seok et al., 2015
AFB1	SERS aptasensor	Electrochemical impedance spectroscopy and SERS	1 × 10^−6^-1 ng mL^−1^	0.4 fg mL^−1^	Peanut	Li et al., 2017
AFB1	SERS aptasensor	Magnetic beads (CSFe3O4) as enrichment nanoprobe and AuNR@DNTB@Ag nanorods (ADANRs)	0.01–100 ng mL^−1^	3.6 pg mL^−1^	Peanut oil	Chen et al., 2018
AFM1	Electrochemical aptasensor	Fe3O4-incorporated polyaniline (Fe3O4/PANi) film	6–60 ng L^−1^	1.98 ng L^−1^	–	Nguyen et al., 2013
AFM1	Electrochemical aptasensor	Carbon screen-printed electrode and ferri/ferrocyanide redox probe	2–150 ng L^−1^	1.15 ng L^−1^	Milk	Istamboulie et al., 2016
AFM1	Microring Resonators aptasensor	Silicon oxynitride (SiON) microring resonators	–	5 nM	–	Chalyan et al., 2017
AFM1	Fluorescent aptasensor	RT-qPCR amplification	1.0 × 10^−4^-1.0 μg L^−1^	0.03 ng L^−1^	Rice cereal, milk powder	Guo et al., 2016
AFM1	Fluorescent aptasensor	Graphene oxide (GO) and nuclease amplification	0.2–10 μg kg^−1^	0.05 μg kg^−1^	Milk powder	Guo et al., 2019
FB1	Electrochemiluminescence aptasensors	Gold nanoparticles (Au NPs) and ionic iridium complex	0.5–50 ng mL^−1^	0.27 ng mL^−1^	Wheat flour	Zhao et al., 2014
FB1	Microcantilever array aptasensor	Array with self-assembled monolayers (SAMs) functionalized sensing cantilevers	0.1–40 μg mL^−1^	33 ng mL^−1^	–	Chen et al., 2015
ZEN	Fluorescent aptasensor	Upconverting nanoparticles	0.05–100 μg L^−1^	0.126 μg kg^−1^/0.007 μg L^−1^	Corn/Beer	Wu et al., 2017

Simple fluorescent aptasensors have been reported for AFB1 determination based on the target-induced structure of switchable aptamers and the high distance-dependent fluorescence quenching property of GO. First, Lu et al. reported a fluorescent aptasensor using quantum dots and GO for fluorescence quenching (Lu et al., 2014). A thiol-functionalized aptamer was modified at the CdTe quantum dots surface via ligand exchange. In the absence of AFB1, the fluorescence signal of the Q-dots was dramatically quenched by GO. Subsequently, fluorescence was recovered upon the presence of AFB1 ranging from 3.2 nM to 320 μM, and its detection limit was 1.0 nM. Based on a similar principle, Zhang et al. introduced an amplified fluorescence aptasensor for AFB1 determination. The aptasensor adopted the ability of GO for aptamer protection from nuclease cleavage and used the nanometer size performance of GO to improve sensitivity. Three detection ranges (12.5–312.5, 5.0–50, and 1.0 to 100 ng mL^−1^) were achieved with a detection limit of 10.0, 15.0, and 0.35 ng mL^−1^, respectively (Zhang et al., 2016a). Furthermore, a colorimetric method was carried out for AFB1 determination using the aptamer and two split DNAzyme halves. A good linear response was achieved between the colorimetric signal and AFB1 concentrations ranging from 0.1 to 1 ng mL^−1^, and the detection limit was 0.054 ng mL^−1^. This described aptasensor demonstrated high specificity for AFB1 and could be applied for AFB1 determination in corn (Seok et al., 2015).

Surface-Enhance Raman Scattering (SERS) is an important analytical technique that is widely used to analyze a large amount of contaminated agricultural products. First, Li et al. reported an aptasensor for AFB1 determination based on SERS signal amplification (Li et al., 2017). In this study, under AFB1 addition, an aptamer/AFB1 complex was formed, resulting in the dissociation of complementary DNA, which was as a signal enhancement element for the next cycle. Under the DNA hybridization, a SERS tag was conjugated on the surface of AuNPs. A good linear response was achieved between the electrochemical signal and AFB1 levels in the range of 1 × 10^−6^ to 1 ng mL^−1^ and with a detection limit of 0.4 fg mL^−1^. Recently, based on the same SERS technique, an ultrasensitive aptasensor for AFB1 determination was introduced using CS-Fe_3_O_4_ nano-bead signal enrichment. Under the addition of AFB1, the release of SH-DNA2-ADANRs from the surface of CS-Fe_3_O_4_ was induced as a result of the competitively binding reaction between AFB1 and NH2-DNA1-CS-Fe_3_O_4_, and the signal of SERS was thus decreased. The electrochemical signal exhibited a linear relationship with AFB1. The concentrations ranged between 0.01 and 100 ng mL^−1^ with a detection limit of 0.0036 ng mL^−1^ (Chen et al., 2018). The aptasensors based on the SERS technique took advantage of greatly improving the detection stability and sensitivity.

**Figure 7:**
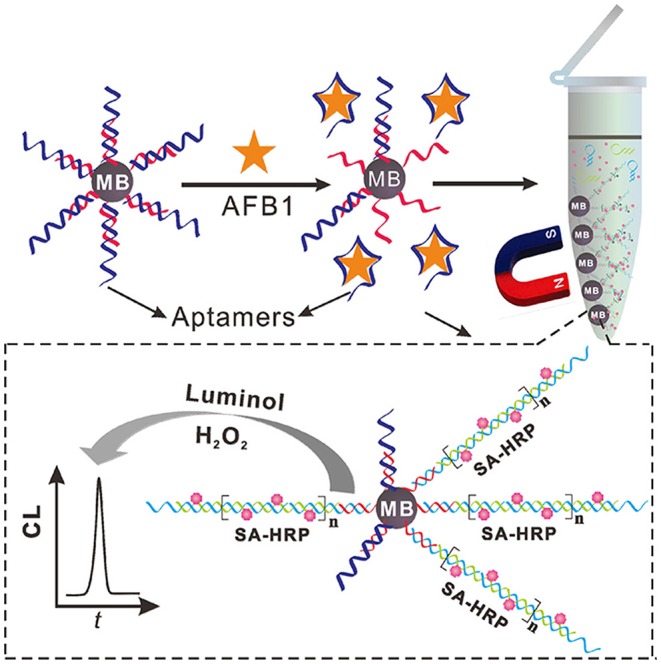
Schematic illustration of chemiluminescent aptasensor for AFB1 determination by using HCR signal amplification. Reprinted from Yao et al. (2019) with permission.

**Figure 8:**
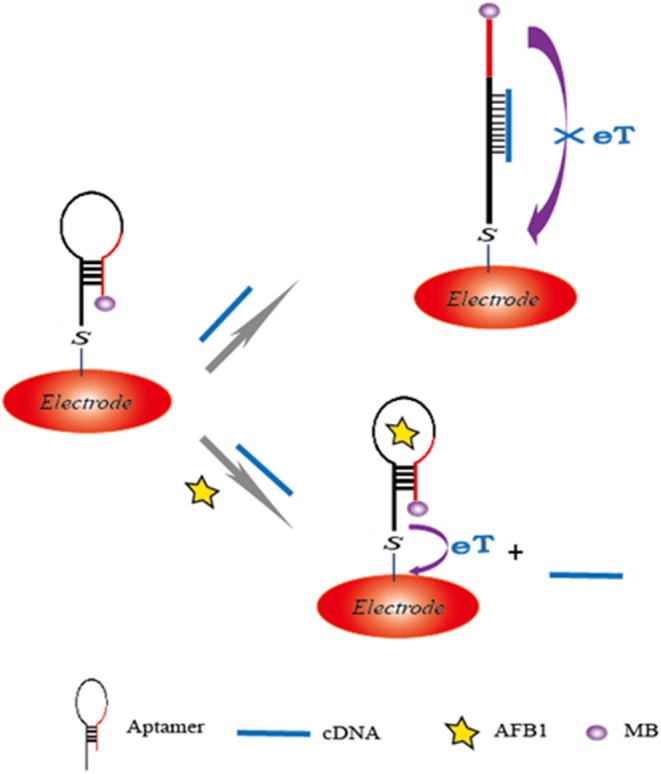
Sensing illustration of electrochemical aptasensor for detection of AFB1 based on the conformational change of aptamer.

The hybridization chain reaction (HCR), a signal amplification strategy, has been widely used to construct biosensors for target determination. In 2019, Yao et al. developed a chemiluminescent aptasensor via HCR for the detection of AFB1 with high sensitivity (Yao et al., 2019). In this design (Figure 7), the aptamer was hybridized with a probe to form dsDNA on the surface of magnetic beads (MBs). When AFB1 existed, the formation of an aptamer/AFB1 complex led to the release of the probe on MBs after magnetic separation. Therefore, HCR signal enhancement and HRP catalysis were achieve with high AFB1 detection sensitivity, and its detection limit was established at 0.2 ng mL^−1^. Based on a similar competitive binding of an aptamer between the target and cDNA, for more sensitive method to meet the requirement of low MRL, Wang et al. introduced a simple electrochemical aptasensor for AFB1 determination with high sensitivity (LOD = 2 nM). The aptamer was labeled with methylene blue (MB) and immobilized on a gold electrode. In the presence of AFB1, the conformational change of the aptamer resulted in the MB moving closer to the surface of the electrode, which caused the increase of the current (Figure 8). More importantly, a rapid aptasensor was achieved, as it only took 5 min to complete the sample incubation (Wang C. et al., 2019). In the pursuit of developing a more rapid on-site sensing strategy for AFB1 determination, Xia et al. reported an ultrafast aptasensor for the detection of AFB1 based on enzyme-free signal amplification and a special aptamer design (Xia et al., 2019). In this work, the aptamers were adopted as dual-terminal proximity structures with two fluorophores. These two fluorophores lit up when the aptamer rapidly recognized one molecule of AFB1. This approach required only 1 min to complete the analysis process, which means it was the fastest method for AFB1 detection until now. Therefore, the proposed aptasensor has a promising application for on-site detection of mycotoxins. The aptamer of AFM1 was first produced using the SELEX technique, which was obtained from the Institute of Biotechnology, Vietnam Academy of Science and Technology. Based on this specific aptamer, Nguyen et al. reported an electrochemical aptasensor for AFM1 determination, which combined the unique recognition ability of aptamers with the signal enhancement function of Fe_3_O_4_ magnetic nanomaterials. Under the condition of this amplification method, a detection limit (1.98 ng L^−1^) was obtained in the detection range of 6–60 ng L^−1^. However, the practical application of this aptasenor for matrices has not been validated, and further studies are thus required on the subject of practical samples toward the inclusion of a large number of mycotoxins for food safety (Nguyen et al., 2013). Nevertheless, an electrochemical impedance biosensor was described for AFM1 determination using the same specific aptamer, and this validation method in milk samples was investigated (Istamboulie et al., 2016). With the production of an aptamer/AFM1 complex, the electrochemical signal was enhanced with the increase of AFM1 concentrations ranging from 2 to 150 ng L^−1^ in buffer, and its detection limit was 1.15 ng L^−1^. In addition, the current method was shown to detect AFM1 in a range of 20–000 ng kg^−1^ in milk, demonstrating that the developed aptasensor was an effective sensing strategy for the analysis of AFM1. Then, optical biosensors based on microring resonators (MRR) have also been used for biomolecular analyses in food safety. A functionalized silicon oxynitride MRR-based biosensor was reported for AFM1 determination. The binding of AFM1 on the functionalized microring surface led to the effective refractive index change, and a resonance shift in the MRR transmission was then detected using off-chip silicon photodetectors. In addition, this approach was demonstrated for AFM1 determination specifically with a detection limit of 5 nM (Chalyan et al., 2017).

**Figure 9:**
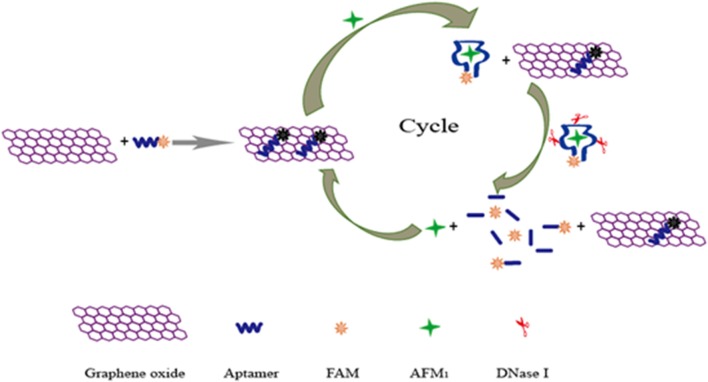
Schematic illustration of fluorescent aptasensor for detection of AFM1 by using graphene oxide signal amplification. Reprinted from Guo et al. (2019) with permission.

Recently, a new aptamer specific to AFM1 was selected by a traditional SELEX technique with the dissociation constant of 35 nM (Malhotra et al., 2014). Based on the high-affinity aptamer, a real-time quantitative polymerase chain reaction (RT-qPCR) sensor was introduced for the detection of AFM1 in our research using target-induced strand displacement (Guo et al., 2016). In this detection method, the aptamer was adopted to recognize target AFM1, and its complementary ssDNA was used as template of RT-qPCR for signal amplification. A good linear response was observed between cycle threshold values and AFM1 levels in the detection range of 1.0 × 10^−4^ to 1.0 ng L^−1^, and its detection limit was 0.03 ng L^−1^. However, complicated instruments and procedures were required in this aptasensor. In order to overcome these difficulties, a rapid fluorescent aptasensor was developed for AFM1 determination in further studies (Guo et al., 2019), which combined the advantages of fluorescence quenching ability of graphene oxide and target-cycled signal amplification induced by DNase (Figure 9). In optimized conditions, this amplified aptasensor showed a good detection range of 0.2–10 μg kg^−1^ for AFM1, and the detection limit was calculated at 0.05 μg kg^−1^. More importantly, the feasibility analysis of this method was successfully carried out in milk powder samples for the detection of AFM1; this aptasensor is a potentially good analytical tool for various mycotoxins for food safety.

## Aptasensors for Other Mycotoxins

Fumonisin B1 (FB1), the most common mycotoxin in cereals, could lead to the development of serious threats, such as cancer, in humans and animals. The aptamer specific to FB1 was first selected by the research team in Canada (McKeague et al., 2010). The dissociation constant was established to be 100 nM, and, after the selection and characterization of the specific aptamer to FB1, an electrochemiluminescence (ECL) aptasensor was introduced for FB1 determination with good sensitivity and high accuracy via the unique recognition between FB1 and the aptamer and excellent conductivity of AuNPs. In this study, the ionic iridium (Ir) complex improves the ECL signal. Its detection limit was calculated at 0.27 ng mL^−1^ (Zhao et al., 2014).

The microcantilever method was used to construct a biosensor for mycotoxin detection. Chen et al. provided an array sensor functionalized by self-assembled monolayers (SAMs) and the thiolated aptamer (Chen et al., 2015). A non-specific response could cause a deflection. To prevent the interference from the environment, 6-mercapto-1-hexanol SAMs were adopted to modify reference cantilevers. The differential cantilever signals showed a good linear response with FB1 levels in the range of 0.1–40 μg mL^−1^ and a detection of 33 ng mL^−1^.

**Figure 10:**
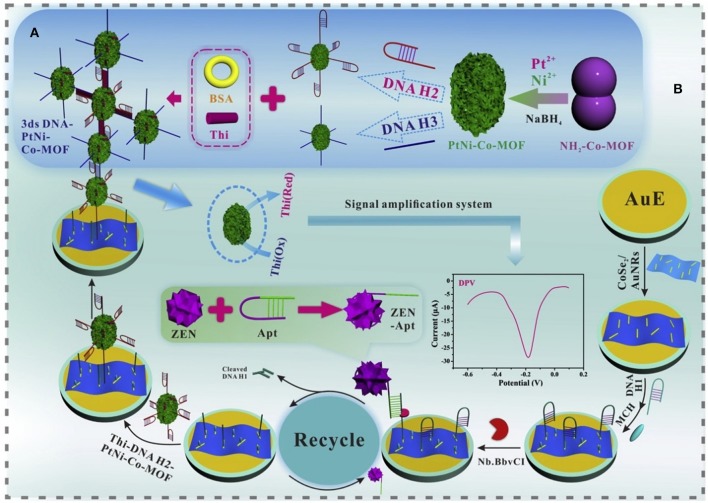
**(A)** Diagram of the construction of 3 ds DNA-PtNi@Co-MOF networks. **(B)** Schematic illustration of the proposed aptasensor for the detection of ZEN. Reprinted from He and Yan (2020) with permission.

Zearalenone (ZEN) was one of the non-steroidal estrogenic mycotoxins, and ZEN and its metabolites were confirmed to have competitive reactions with estrogen receptors. Due to its widespread existence in corn, ZEN posed great hazards to humans and animals and affected the quality of corn products (Yazar and Omurtag, 2008). The specific aptamer to ZEN was selected and characterized by Wang's research team (Chen et al., 2013). Up to now, there have been few studies into aptasensors for ZEN determination. Nevertheless, in 2017, a fluorescent aptasensor, created through upconversion nanoparticles, was successfully developed for ZEN determination in corn and beer (Wu et al., 2017). The specific aptamer to ZEN was employed as a recognition probe while the complementary strand was adopted as a signal probe. A linear relationship was achieved between the luminescence signals and ZEN concentrations ranging from 0.05 to 100 μg L^−1^. Its detection limit was determined at 0.126 μg kg^−1^ in corn and 0.007 μg L^−1^ for beer, demonstrating that the developed aptasensor offered a novel approach for the application analysis of ZEN for food safety. Very recently, an ultrasensitive electrochemical aptasensor was achieved for the detection of ZEN with higher sensitivity (LOD = 1.37 fg mL^−1^). CoSe2/AuNRs, PtNi@Co-MOF, and nicking enzyme were adopted to construct the sensing platform (Figure 10). Through this experimental design, a significant signal amplification was obtained with good stability and specificity, which indicated that this proposed aptasensor strategy offered a promising application to quantify even trace levels of ZEN for food safety (He and Yan, 2020).

## Aptasensors for Simultaneous multi-Mycotoxins Analysis

Hundreds of mycotoxins occur and are identified in food and agricultural products (Silvério et al., 2010; Deng et al., 2013), and the coexistence of multiple mycotoxins is a complex phenomenon. However, numerous articles published have mainly aimed toward the single detection of mycotoxin. Therefore, the development of novel aptasensors for simultaneous analysis of multi-mycotoxins is very important in mycotoxin analyses for food safety.

Recently, a novel and rapid electrochemical aptasensor for the simultaneous analysis of two mycotoxins (OTA and FB1) was developed via the specific recognition of an aptamer and targets (Wang et al., 2017). In this work, CdTe and PbS QDs acted as the marks while these complementary DNA sequences were employed as the interaction probes. Upon the addition of target OTA and FB1, the interaction of the aptamer and targets caused the release of QDs. After magnetic separation, good linear responses were determined to be between the electrochemical signals and target concentrations in a dynamic range of 0.01–10 ng mL^−1^ for OTA and 0.05–50 ng mL^−1^ for FB1. In addition, this electrochemical aptasensor method has been successfully used for these two targets in maize samples. Therefore, the proposed method offered a promising strategy for multiple mycotoxin analysis for food safety.

Moreover, in addition to a quantum dots (QDs)-labeled electrochemical method, fluorescence aptasensors based on photonic crystal microsphere suspension technique could also apply to the multiplex mycotoxin detection. Yang et al. introduced a potential fluorescent aptasensor for high-throughput detection of multiple mycotoxins via a PHCM suspension array. In target mycotoxins addition, the conformation of a aptamer/target complex resulted in changes in the fluorescent signals, which had linear responses with targets levels in the same range of 0.1 pg mL^−1^ to 0.1 ng mL^−1^ for OTA and AFB1 and 0.1 to 10 ng mL^−1^ for FB1. The limits of detection were established to be 3.96 fg mL^−1^ for OTA, 15.96 fg mL^−1^ for AFB1, and 11.04 pg mL^−1^ for FB1 (Yang et al., 2017).

In addition, DNA-scaffolded silver nanocluster and magnetic separation could also be used for simultaneous determination of multi-mycotoxins. Zhang et al. introduced a highly sensitive aptasensor for the simultaneous analysis of AFB1 and OTA. In this design, two ssDNA were hybridized with two aptamers specific to these two mycotoxins, which were employed for the synthesis of silver nanoclusters. Upon the addition of OTA and AFB1, the release of these two probes was induced due to the structure change of the aptamers. After magnetic separation and the subsequent synthesis of silver nanoclusters, the significantly increased fluorescence intensity was achieved and determined to display a strong linear relationship for both these two mycotoxins in the same range of 0.001–0.05 ng mL^−1^, and the detection limits of AFB1 and OTA were established at 0.3 and 0.2 pg mL^−1^, respectively. Moreover, the practical application of this fluorescence aptasensor was investigated via the analysis of real cereal samples, demonstrating that the proposed approach might represent a potential sensing strategy for multiple mycotoxins determination (Zhang et al., 2016b).

**Figure 11:**
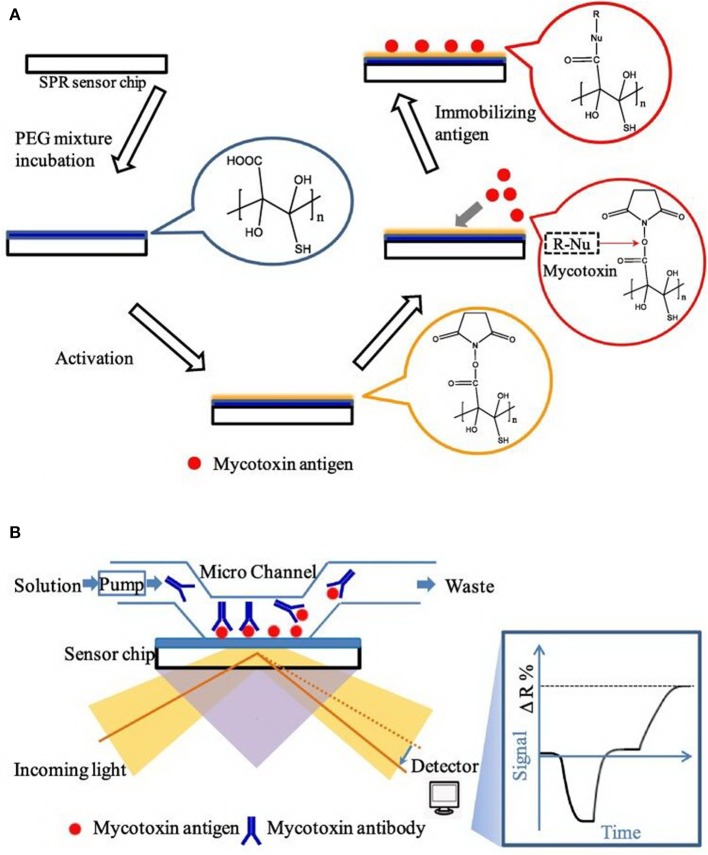
**(A)** Principle diagram of SPR aptasensor platform. **(B)** Principle diagram of sensor chip and optical setup in SPR. Reprinted from Wei et al. (2019) with permission.

Finally, surface plasmon resonance (SPR), an important label-free analytical strategy, could be applied for the simultaneous sensitive detection of multiple targets with superiorities, such as good specificity, real-time monitoring, and high throughput detection (Patel et al., 2017). More recently, Wei et al. developed a SPR-based aptasensor chip for the simultaneous determination of four mycotoxins with low crossreactivity (Figure 11), which has been successfully used for practical analysis in wheat and corn. The detection limit for AFB1, OTA, ZEN, and deoxynivalenol (DON) were established at 0.59, 1.27, 7.07, and 3.26 ng mL^−1^, respectively (Wei et al., 2019).

## Challenges and Limitations of Aptasensors

Although those promising advantages of rapid, portable, low-cost, specific, and sensitive characteristics were proven, nucleic acid aptamer-based biosensors still have several key scientific limitations and challenges: (i) as small molecules, the screening of nucleic acid aptamers specific to mycotoxins is a complex and time-consuming process. There are many factors that influence aptamer selection efficiency. Therefore, the successful selection of an aptamer is difficult. Today, only a few nucleic acid aptamers against mycotoxins such as OTA (Cruz-Aguado and Penner, 2008), AFB1 (Patent:PCT/CA2010/001292, Patent:PCT/CA2010/001292) and fumonisin B1 (McKeague et al., 2010) have been successfully screened and applied, and the aptamer of ZEN (Chen et al., 2014) was screened in 2013 and has not yet been applied for practical analysis. There are hundreds of mycotoxins that have been identified, demonstrating that the available aptamers far from meet the requirement of mycotoxin detection. (ii) Some mycotoxins have various structural analogs, such as aflatoxin, which contains B1, B2, M1, M2, G1, G2, etc. It is more difficult to select the highly specific aptamers for each analog. The method behind identifying and distinguishing between similar mycotoxins is also important. Therefore, the bottleneck problem on the development of mycotoxin aptasensors is how to identify and detect multiple mycotoxins using the limited variety of mycotoxin aptamers, especially for structurally similar mycotoxins.

## Conclusions and Future Trends

In this review paper, it was demonstrated that a series of aptasensors were successfully used for the determination of small molecules mycotoxins. Current reports illustrated here indicated the high promising benefits of the aptamer-based biosensors for mycotoxins determination owing to their rapid and sensitive analysis in foods and agricultural products. In comparison with traditional instrumental approaches, including HPLC with fluorescence detection (FLD) and HPLC-MS, aptasensor techniques have the advantages of rapid, portable, and low-cost targets analysis as well as having great potential for field determination and high-throughput identification of multiple mycotoxins. In addition, taking mycotoxin hazards into consideration, aptasensor strategies provides high sensitivity and selectivity for mycotoxin determination to meet the requirement of maximum limits set by many countries and regulators.

Aptamers have been considered to be “chemical antibodies” that are capable of recognizing and binding targets with high affinity and selectivity that are similar or even superior to antibodies. With these unique advantages, aptasensors have been widely developed for the detection of mycotoxins and have attracted more and more attention from researches. However, for practical samples, such as food and agricultural products, antibody-based immunoassays are mainly used, while the application of aptamer-based biosensors in the detection of mycotoxins is still in varying stages of scientific research. Up until now, no commercial kit based on aptamers has been produced and applied. In our opinion, there are several reasons for this. First of all, traditional antibody preparation needs animal experiments and is thus difficult to prepare in large quantities through chemical synthesis, and antibody preparation technology can also be monopolized due to technical barriers. However, once the aptamer is screened successfully, the sequence of aptamer will be disclosed and can be prepared in large quantities through chemical synthesis, which is difficult to form technical barriers for commercial companies. Secondly, the affinity of aptamers is generally weaker than that of antibodies. In the application of complex systems, affinity and sensitivity are always a challenge. Third, commercial kits need to consider sensitivity, stability, repeatability, cost, technical barriers, and other factors. Currently, the published scientific papers on the detection of mycotoxins by aptamer biosensors may be superior to the ELISA of antibody technology in some aspects, such as sensitivity, stability, repeatability, time consuming, cost, etc., but the comprehensive performance is not necessarily superior to an ELISA. There are few articles comparing aptamer-based biosensors with a commercial ELISA kit that takes into consideration all these aspects. Limitations of aptamers are mainly, though not limit to, the requirements for sophisticated instruments, professional personnel, extraneous signal interference, time spent, as well as quality of aptasensor systems. Future direction will focus on the simplification of analytic principle and devices and the combination of novel aptamers with new materials and techniques to improve the analytical performance and market practicality of aptasensors.

There are hundreds of mycotoxins occurring in food and agricultural commodities, and the coexistence of multiple mycotoxins is a very common phenomenon. However, numerous research into the present literatures focus on the single analysis of OTA and AF detection. Therefore, future research is required for the successful selection of more specific aptamers against other mycotoxins and the development of novel aptasensors and sensor arrays for multi-mycotoxin analyses. In the future, aptamer-based biosensors will greatly contribute to the safety control in food and agricultural products or other research fields.

## Author Contributions

All authors listed have made a substantial, direct, and intellectual contribution to the work and approved it for publication.

## Conflict of Interest

The authors declare that the research was conducted in the absence of any commercial or financial relationships that could be construed as a potential conflict of interest.
